# Effect of Axial and Coronal Malalignment on Polyethylene Stresses in Lumbosacral Total Joint Replacement: A Finite Element Study

**DOI:** 10.3390/bioengineering13090987

**Published:** 2026-08-27

**Authors:** Steven A. Rundell, Steven M. Kurtz, Hannah Spece, Scott D. Hodges, Ron V. Yarbrough

**Affiliations:** 1 Explico Inc., Detroit, MI 48375, USA; 2 Implant Research Core, School of Biomedical Engineering, Science and Health Systems, Drexel University, Philadelphia, PA 19104, USA; 3 Gyroid LLC, Haddonfield, NJ 08033, USA; 4 Spine Motion Specialists, Chattanooga, TN 37402, USA; 5 3Spine Inc., Chattanooga, TN 37402, USA

**Keywords:** lumbar total joint replacement, spine, finite element analysis, polyethylene wear, contact stress, misalignment

## Abstract

The purpose of this study was to analyze the sensitivity of polyethylene stresses and strains, as proxies for wear and surface damage, to coronal and axial misalignment for a novel lumbar total joint replacement (LTJR) implanted at L5-S1. We hypothesized that these stresses and strains would remain below the levels associated with worst-case impingement in a spine wear simulator. A finite element model (FEM) of the L4-S1 spine was developed using CT-based anatomy from a representative Investigational Device Exemption study patient. An appropriately sized LTJR was virtually implanted at L5-S1 in a standardized baseline orientation, and component positioning was altered to achieve eight independent misalignment scenarios, spanning axial convergence angle, anterior–posterior offset, and coronal tilt. Physiologic forward-bending loads were applied, and polyethylene contact pressure, von Mises stress, and effective strain were recorded. Peak values for the baseline scenario were 35.0 MPa, 19.9 MPa, and 4.8%, respectively. Convergence angle and anterior–posterior offset had minimal effect, whereas coronal tilt produced the largest changes. Contact pressure and von Mises stress varied by <20% due to misalignment, whereas effective strain was more sensitive, increasing from 4.8% at baseline to 9.9% with 20 degrees of coronal tilt. All values were comparable to prior L4-L5 findings and remained well below impingement levels. Within the bounds of reasonable misalignment, the device maintained bearing congruency and did not approach conditions associated with accelerated wear.

## 1. Introduction

Symptomatic degenerative disc disease (DDD) of the lumbar spine can result in a substantial reduction in the quality of life. Although the current gold standard treatment continues to be lumbar spinal fusion, the development of adjacent level disease, which may be in part attributed to an altered biomechanical environment, has been observed clinically and in in vitro studies [1,2,3,4,5]. Historically, total disc replacement systems (TDR) have been proposed as an alternative to fusion. These devices are intended to restore disc height, maintain or correct segmental lordosis, and preserve segmental range of motion [6]. Biomechanical studies have documented a reduction in adjacent level effects after TDR when compared with fusion [5,7]. However, some potential complications associated with TDR include misalignment of the devices, resulting in excessive polyethylene (PE) stresses and consequent wear. Specifically, device impingement has been observed clinically [8,9,10], and excessive PE wear with associated osteolysis has been reported in a small number of cases [11,12,13,14,15]. These findings highlight the importance of understanding the long-term clinical wear performance of lumbar total disc and joint replacements, particularly given their frequent use in young, active patients [16].

The MOTUS^®^ lumbar total joint replacement (LTJR) is a novel device under evaluation in an Investigational Device Exemption (IDE) clinical trial as a condition of Premarket Approval by the United States Food and Drug Administration [17]. Unlike traditional disc replacements, the LTJR design addresses the biomechanics of both the disc and the facets with its novel dual ball-and-socket design, which constrains the functional spinal unit predominantly to motion about the flexion–extension axis [18,19,20]. The dual bearing LTJR design comprises two sets of CoCr alloy caudal components articulating with metal-backed highly crosslinked and Vitamin E stabilized polyethylene cranial components [21]. The device has undergone preclinical wear testing in both normal and impingement wear modes, exhibiting comparable or less wear than anterior lumbar disc replacements [21].

As an investigational device, little is known about its sensitivity to coronal and axial plane alignment when implanted. We first examined this question using a computational model of the LTJR in the context of a spine wear simulator [22]. In a subsequent study, we virtually implanted the LTJR into the L4-L5 disc space of a L3-L5 spine model that had been previously validated in the literature [23]. However, the LTJR has a broader indication than L4-L5, with the IDE study indicated for reconstruction of a single level between L1-L2 and L5-S1 [17]. At present, approximately half of the patients of the fully enrolled clinical trial have been treated at L4-L5, with the remaining half treated at L5-S1.

Notably, the L5-S1 functional spinal unit presents biomechanical considerations that are distinct from more cranial levels. As the most inferior segment of the lumbar spine, L5-S1 bears the greatest compressive load from body weight. Additionally, L5-S1 has a distinct range-of-motion profile compared to upper levels [24], and sacral slope presents uniquely pronounced anterior loading [25,26,27]. These differences preclude direct extrapolation of our earlier misalignment sensitivity findings, and the question therefore remains whether the lessons learned from modeling implant misalignment in a spine wear simulator [22] and at a treated level of L4-L5 [23] will be relevant at L5-S1.

The purpose of the present study was to analyze the sensitivity of the polyethylene stresses and strains associated with wear and surface damage in the LTJR to coronal and axial misalignment at L5-S1. We hypothesized that the polyethylene stresses and strains, albeit higher under the greater segmental loading than our previous L4-L5 treated model, would similarly remain lower than the levels previously encountered during worst-case impingement loading conditions in a spine wear simulator [22]. To test this hypothesis, we built a lumbosacral finite element model (FEM) based on clinically representative anatomy and virtually implanted an appropriately sized LTJR at L5-S1.

## 2. Methods

### 2.1. Model Description

The LTJR Spine L4-S1 FEM consists of a virtually implanted LTJR device in the lumbar spine at L5-S1. The current model was built on the previously verified and validated standalone LTJR device FEM [22,28] and uses the same approach to verification and validation as a previous LTJR L3-L5 Spine FEM [23,29]. The credibility of the integrated L4-S1 model was established in accordance with ASME V&V 40:2018 [30] and 2023 FDA guidance [31], as reported in detail in a companion study [32]. Briefly, the level of rigor was set according to the model’s low-risk classification. Verification addressed software and code accuracy (benchmarked within 5% of Ansys LS-DYNA verification cases), mesh discretization (a 0.6 mm element size yielding contact pressures that converged within 1%), quasi-static behavior (negligible kinetic energy throughout each simulation and applied, muscular, and bearing reaction forces balanced within a few percent), and independent internal review of material property assignments, boundary conditions, contact definitions, and outputs. Validation confirmed that the model behaved consistently with the independently validated device and lumbar sub-models and produced physiologically appropriate forces [32].

Unlike the previous L3-L5 model, which was based on anatomic geometry from the literature, the vertebra and sacrum anatomy in the present L4-S1 model was based on the screening CT dataset of a representative patient from the MOTUS^®^ IDE study (G220133) who was implanted with a device at L5-S1. The L4-S1 anatomic bony geometry and L4-L5 intervertebral disc were segmented using DICOM to Print software (version 1.0.5.1000, D2P: Oqton Inc., Ghent, Belgium) and post-processed (e.g., smoothing) using Geomagic Freeform software (version 2024.2.0, Hexagon AB, Stockholm, Sweden). The 3D models of the L4-S1 vertebral bodies and the L4-L5 intervertebral disc were exported for meshing using ANSYS’s WorkBench software (version R15, Ansys, Inc., Canonsburg, PA, USA). The segmentation workflow was validated against independent reference geometries, specifically lumbar vertebral bodies from the Cancer Imaging Archive [33], with a mean dimensional difference of 0.22 ± 0.64 mm, confirming that the CT-based reconstruction reproduced vertebral size and shape with sub-millimeter accuracy [32]. The properties and attachment points for the muscles and ligaments were based on the existing L3-L5 FEM, with changes made as described below. The superior and inferior surface nodes of the discs were rigidly affixed to the corresponding vertebral body surface.

**Figure 1:**
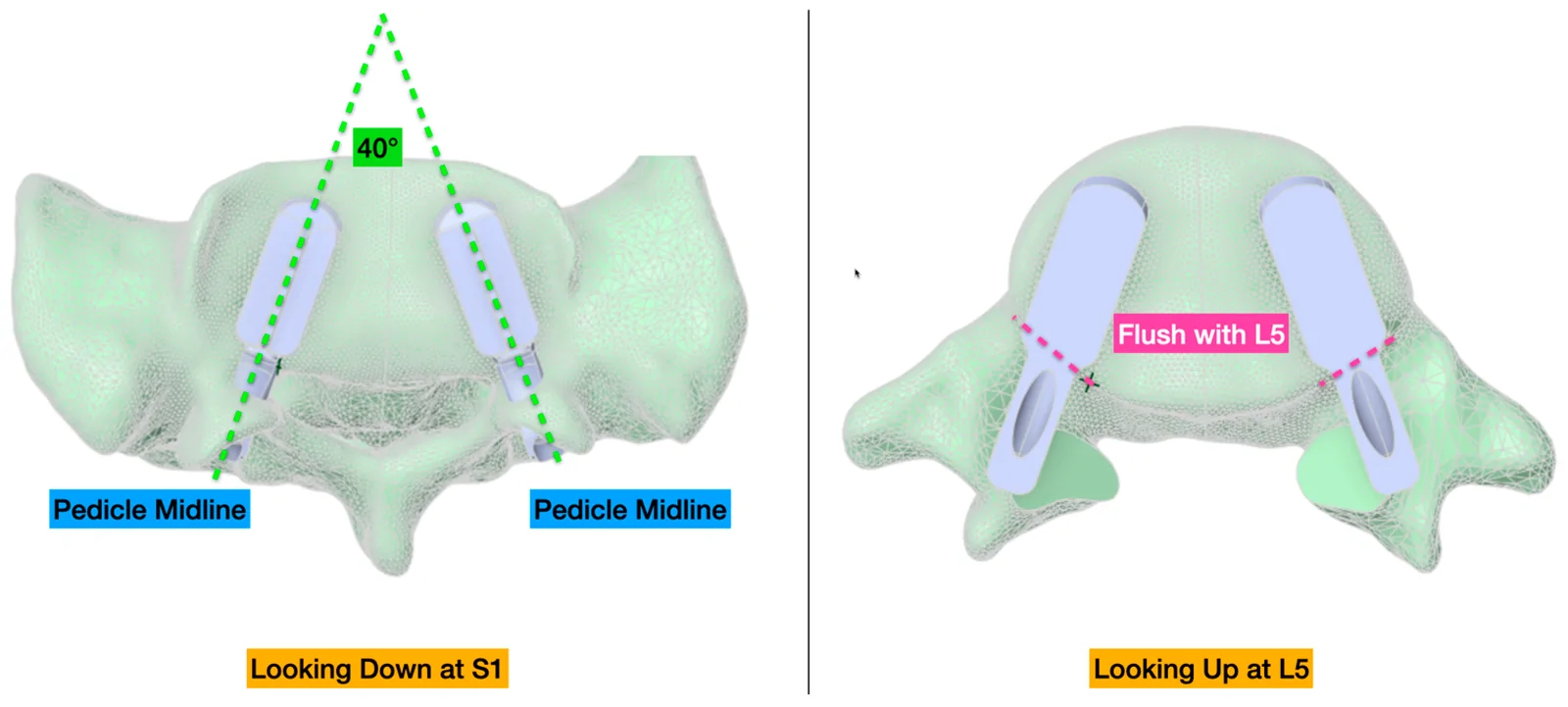
Images depicting the placement and orientation of the LTJR relative to S1 (**left**) and L5 (**right**).

A virtual implantation of the LTJR device components, with the size based on the IDE subject, was performed within ANSYS Workbench. Medical imaging, including six-week post-implantation X-rays and CT scans, served as anatomical guidance to inform implant positioning and spinal segment distraction. While these clinical data provide qualitative alignment with the post-operative configuration, the implant was ultimately positioned according to standardized “baseline” orientation as previously described (convergence angle of 40 degrees, anterior–posterior offset of 0 mm, and coronal tilt of 0 degrees) [22,23]. The final positioning was further governed by midline alignment of the implants with respect to the S1 pedicles and posterior placement such that the posterior-most surface of the superior endplate component was positioned as flush as possible with the posterior margin of the L5 vertebral body (Figure 1).

The L5 vertebra was virtually distracted relative to S1, with the magnitude and direction of distraction based on post-operative radiographic imaging performed as part of spondylosis correction. This reflected a total distraction of 9 mm along the cranial–caudal axis to mimic physiologic changes made to address stenosis due to the collapsed disc and approximately 5 degrees of sagittal extension of L5 for lordotic correction. Alignment of the spine was carried out with respect to the vertical and horizontal axes to ensure loading occurred in a physiologically relevant manner and reflects anatomic changes (introduced via posterior vertebral body osteotomy) to facilitate improved sagittal balance performed by the implanting physician. Specifically, the angle between the implant inferior endplate and the horizontal was set to 40 degrees, based on the average sacral slope as previously reported [34].

**Figure 2:**
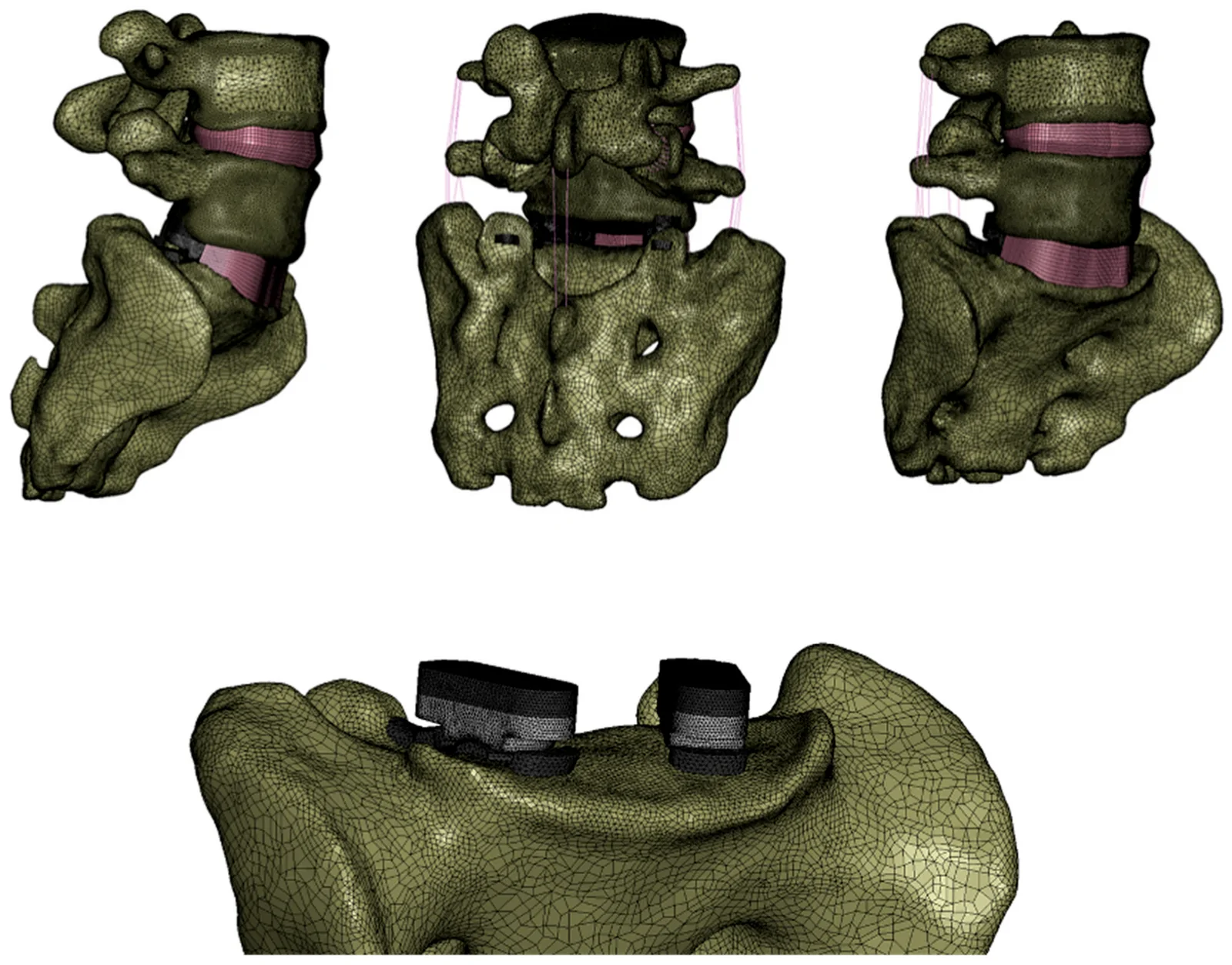
Side, rear, and front views of the MOTUS^®^ Spine L4-S1 FEM as well as an isolated image of the mesh of the device and sacrum. The pink portion in the intervertebral space corresponds to the annulus fibrosus, and for each MOTUS^®^ device the white corresponds to polyethylene and the dark gray to the metallic endplates.

The posterior longitudinal ligament, the supraspinous ligament, the intraspinous ligament, the ligamentum flavum, the facet capsular ligaments, and facets were removed at L5-S1, consistent with the appropriate surgical technique reflecting an open surgical exposure using a posterior midline approach. In general, the posterior elements at L5 were largely removed along with any ligaments or muscles attaching at that location. The iliolumbar ligament was added and attached between the transverse process of L5 and the ala sacralis, which is expected to be intact as described in the surgical technique. All other aspects of the lumbar spine modeling, including the material properties of the spine tissues and the facet contact formulations at L4-L5, were the same as those previously described [23] (Figure 2).

Semi-automated meshing techniques were applied to discretize the LTJR components, the intact disc at L4-L5, and the anterior and lateral portions of the annulus fibrosus, which were maintained according to the surgical technique at L5-S1. Element sizing was determined based on prior mesh convergence and discretization studies [22,23]. The contacting surfaces of the implant and the bony endplates were computationally bonded by constraining corresponding surface nodes to maintain position and simulate idealized fixation. This resulted in nominal geometric overlap between the metallic implant endplates and vertebral bodies. There was no attempt to alter the bony geometry to have it be flush with the implant surface, such as would be the case in a real-world clinical setting, given that both objects were modeled as rigid. This simplification was done to improve computational efficiency without compromising the outcome of interest, that is, the contact pressure at the polyethylene bearing surfaces as well as the internal stress and strain of the polyethylene.

### 2.2. FEM Loading Conditions

Loading conditions were informed by the Three-Dimensional Static Strength Prediction Program (3DSSPP, Version 7.1.3), which has been previously shown to result in static strength and low back forces consistent with physiological loading for common workplace postures [35,36,37]. Specifically, a 3DSSPP analysis for forward bending for a 50th percentile adult male was performed in order to determine the appropriate boundary conditions.

The sacrum was fully constrained in all six degrees of freedom (zero displacement and zero rotation in all directions). No kinematic constraints were applied to L4 or L5, which were free to translate and rotate under the applied loading. A Cartesian coordinate system was used throughout the model, with the *x*-axis directed laterally to the right, the *y*-axis directed anteriorly, and the *z*-axis directed inferiorly.

**Figure 3:**
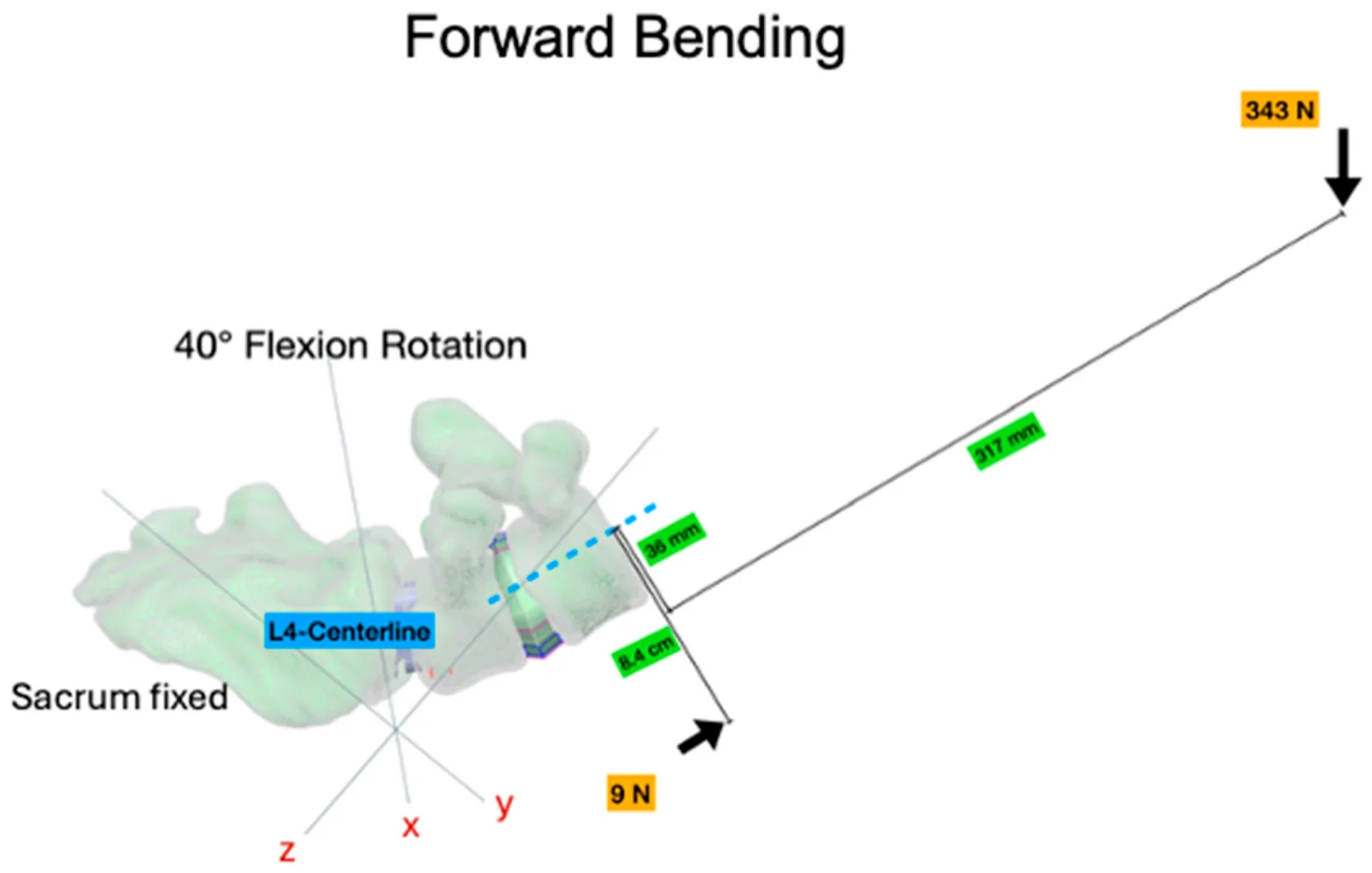
Schematic (not to scale) depicting forward bending boundary conditions. The sacrum is fixed in all degrees of freedom.

A load of 343 N was applied at a node located 36 mm anterior and 317 mm superior to the center of the L4 vertebra to represent upper body weight [38] (Figure 3). The force was decomposed into a caudally directed component (z-direction) of 60 N and an anteriorly directed component (y-direction) of 338 N. These nodes were computationally considered part of the rigid L5 vertebral body. Consequently, forward bending was produced through the eccentricity of this applied load relative to the spinal segments, rather than through application of a bending moment. A counteracting AP force of 9 N in the negative z-direction was also applied. No follower load was employed in this model. Although not an applied load, the muscle reaction forces at both the spinous and transverse processes reach whatever magnitude is necessary to balance intersegmental flexion rotation.

### 2.3. Geometry and Configuration of Reasonably Misaligned Models

**Figure 4:**
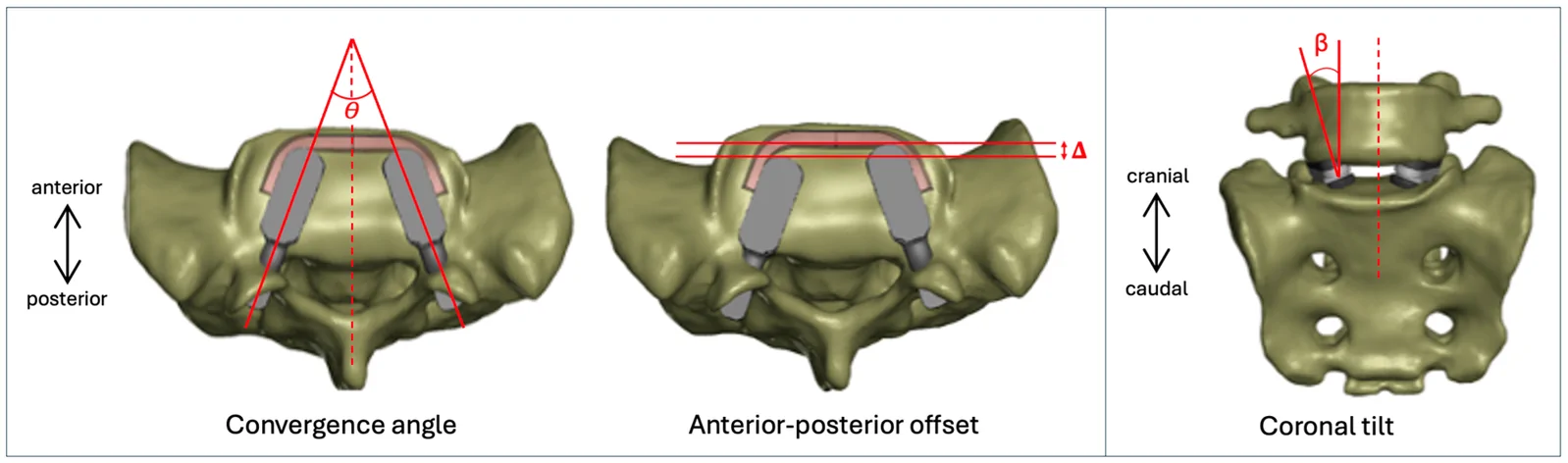
Images of the implanted FEMs illustrating how component misalignment is defined for convergence angle and anterior–posterior offset, viewed in the axial plane, and positive coronal tilt, viewed in the coronal plane. Dotted lines represent the vertebral body midline.

For each analysis, the LTJR component positioning was altered from the baseline condition to achieve one of eight independent misalignment scenarios for axial plane convergence angle, axial plane anterior–posterior (AP) offset, and coronal plane tilt (Figure 4). Convergence angle refers to the angle between the component midlines in the axial plane, with a baseline value of 40 degrees. Misalignment cases of 30 degrees (Case 1) and 20 degrees (Case 2) were evaluated. AP offset refers to the anterior translation of one bearing pair relative to the contralateral pair, which remains in its baseline position. The baseline condition corresponds to 0 mm AP offset. Misalignment cases of 2 mm offset (Case 3) and 4 mm offset (Case 4) were evaluated. Coronal tilt refers to the angular rotation of the implant with respect to the vertebral body longitudinal axis (positive for external rotation and negative for internal rotation), with a tilt of 0 degrees for the baseline condition. Coronal tilts of −20, −10, +10, and +20 degrees were evaluated (Cases 5 through 8). The magnitude of these perturbations was informed by the criteria for “unreasonable misuse” in the surgical technique guide. In total, nine simulations (baseline + each misalignment scenario) were conducted.

### 2.4. Comparison with Previous Models

For each analysis, the contact pressure, von Mises stress, and effective strain were recorded. The results were compared to those previously reported for our standalone device FEM, which models the LTJR devices under Mode I (normal) and Mode IV (impingement) wear conditions in a spine simulator [22]. Importantly, this model was validated against real-world bench testing [21], during which the LTJR performed in a reasonable and expected manner, even under worst-case impingement loading. Thus, the stresses and strains reported for the standalone FEM under Mode I and Mode IV conditions provide important context and benchmarking for our current model. Results from the current study were also compared to the previous L3-L5 FEM [23].

## 3. Results

### 3.1. Convergence Angle

**Figure 5:**
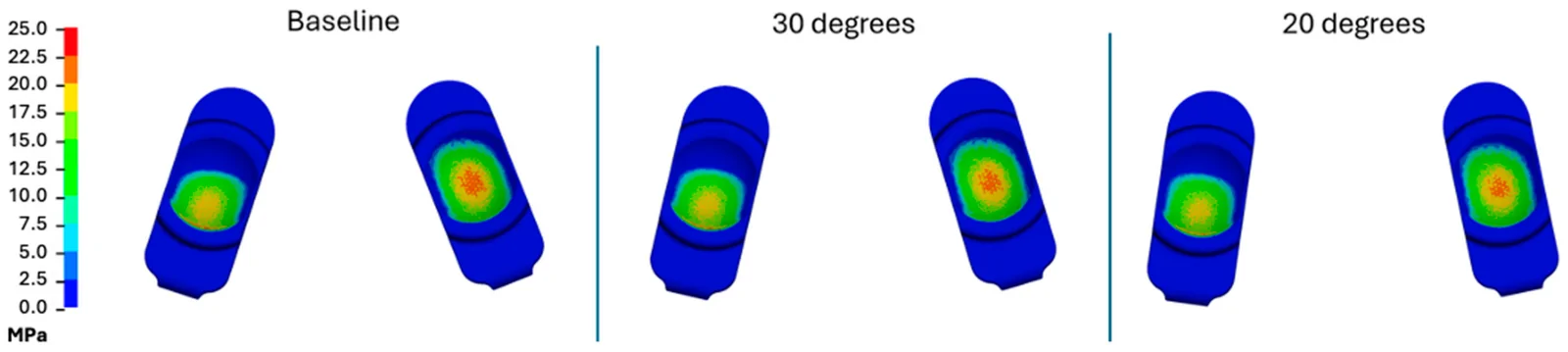
Contact pressure (MPa) plots at maximum loading during bending over for the baseline, 30 degrees, and 20 degrees convergence angle simulations for the L4-S1 FEM. The contour plots are viewed on the MOTUS^®^ superior polyethylene components from the bottom looking up. The up direction in the figure corresponds to the anterior direction.

In general, the convergence angle had a minimal effect on the magnitudes and patterns of contact pressure during forward bending (Table 1, Figure 5). Peak contact pressures for the baseline scenario were 35.0 MPa, decreasing to 34.0 and 32.5 MPa for convergence angles of 30 degrees and 20 degrees, respectively. Similarly, the resulting magnitudes and distributions of von Mises stress and effective strain were generally consistent with the baseline scenario (Supplementary Tables S1 and S2). For von Mises stresses, the peak values were approximately 20 MPa for all scenarios. The effective strain remained at approximately 4.5% for all convergence scenarios.

### 3.2. Anterior–Posterior Offset

**Figure 6:**
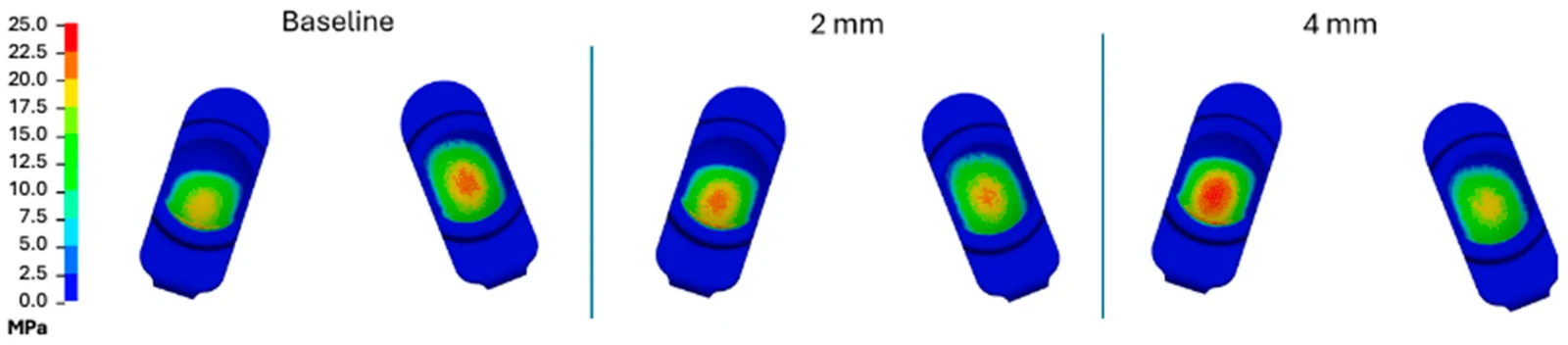
Contact pressure (MPa) plots at maximum loading during bending over for the baseline, 2 mm AP offset, and 4 mm AP offset simulations for the L4-S1 FEM. The contour plots are viewed on the MOTUS^®^ superior polyethylene components from the bottom looking up. The up direction in the figure corresponds to the anterior direction.

In general, contact pressures tended to increase as the component shifted anteriorly (Table 1, Figure 6). Regardless, the general pattern of contact pressure was similar between the scenarios, with all areas of contact pressure restricted to the bearing surface and occurring toward the center of the implant with a slight bias posteriorly. Contact pressures increased negligibly from baseline (35 MPa) to 35.9 at 2 mm offset and 36.6 MPa at 4 mm offset. Results were similar for von Mises stress and effective strain, with slightly greater stress and strain occurring with the anteriorly shifted component (Supplementary Figures S3 and S4). The peak von Mises stress and effective strain were 19.9 MPa and 4.8% at baseline, 20.1 MPa and 5.6% at 2 mm, and 20.9 MPa and 6.6% at 4 mm offset (Supplementary Tables S1 and S2).

### 3.3. Coronal Tilt

**Figure 7:**
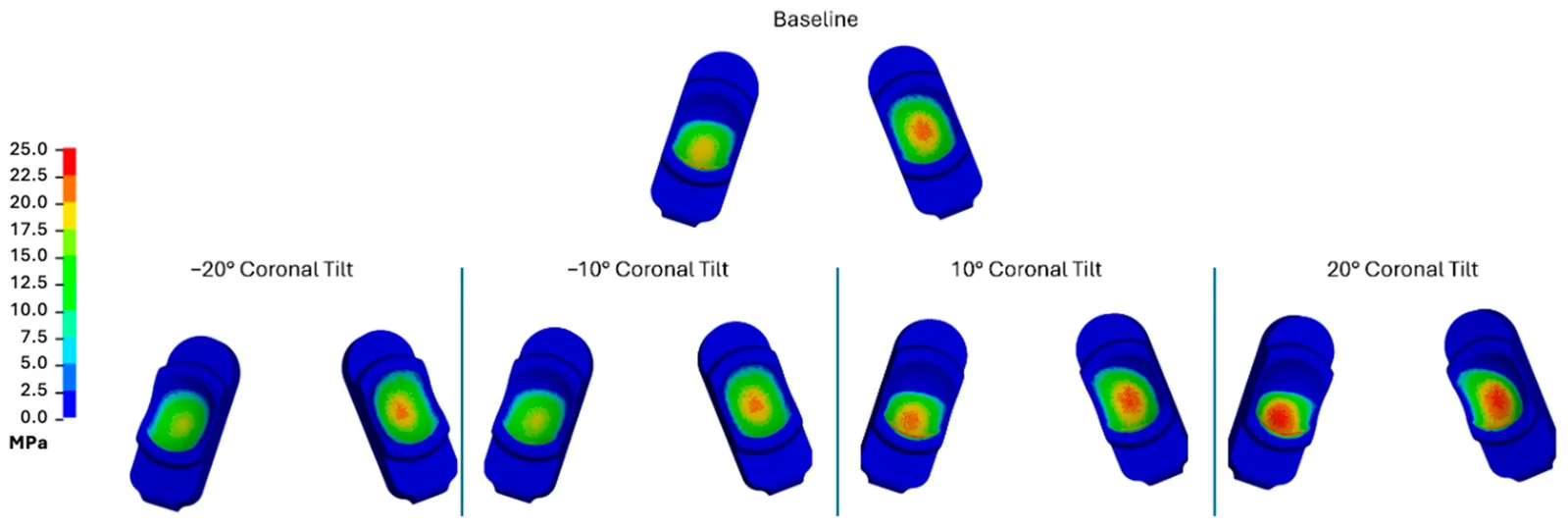
Contact pressure (MPa) plots at maximum loading during bending over for the baseline, −20 degree, −10 degree, 10 degree, and 20 degree coronal tilt simulations for the L4-S1 FEM. The contour plots are viewed on the MOTUS^®^ superior polyethylene components from the bottom looking up. The up direction in the figure corresponds to the anterior direction.

Changes in coronal tilt resulted in the largest impacts on contact pressure (Table 1, Figure 7). Negative coronal tilt angles tended to reduce the peak contact pressures and contours. Conversely, both peak contact pressure magnitudes and contours increased during positive coronal tilt. The increase in contact pressures at the positive coronal tilts coincided with movement of the location of loading away from the center of the domed surface. Peak contact pressure at the baseline scenario was 35.0 MPa. This increased to 36.5 and 36.4 MPa for 10 and 20 degrees of coronal tilt, respectively. The peak contact pressures decreased to 28.2 and 28.8 MPa for coronal tilts of −20 and −10 degrees, respectively. Consequently, the current modeling efforts indicated that positive coronal tilt resulted in the greatest increase in contact pressure during forward bending when compared to the baseline scenario.

Similar differences were observed for von Mises stress and effective strain (Supplementary Tables S1 and S2). The baseline peak von Mises stress was 19.9 MPa, increasing to 20.9 MPa and 22.4 MPa with positive coronal tilts of 10 and 20 degrees, respectively. These values decreased to 19.4 MPa and 19.3 MPa during negative coronal tilts of 10 and 20 degrees, respectively. For effective strain, positive coronal tilt increased the peak value from 4.8% at baseline to 6.8% and 9.9% for 10 and 20 degrees, respectively, while negative coronal tilt reduced the effective strain to 4.2% and 4.1% for −10 and −20 degrees, respectively.

### 3.4. Comparison with Previous Models

The summarized results of the current study are provided in Table 1 along with the results of the previous simulator and L3-L5 FEMs. Similar tables for von Mises stress and effective strain are provided in the Supplementary Materials. The L4-S1 model generally exhibited higher peak contact pressures when compared with the L3-L5 FEM, which is expected given the increased axial loading at this lower spinal level. Additionally, the location of maximum stress in the L4-S1 model was more centrally positioned on the device. The peak values for both von Mises stress and effective strain were lower for the L4-S1 FEM when compared with the L3-L5 FEM for all but one misalignment scenario. This may be due to the more centrally located loading location apparent in the L4-S1 model, or to anatomic variation between models, as boundary conditions and material assumptions were held largely consistent. Notably, the inclination of the sacral endplate alters the orientation of the applied load relative to the bearing, shifting the contact pattern and resulting stress distribution even under equivalent loading. Anatomical differences in endplate geometry may contribute as well, and the assignment of uniform soft tissue properties across levels represents a simplification that may itself influence the comparison. For each case, the peak values remained considerably lower than those associated with Mode IV wear testing conditions (i.e., 83.3 MPa contact stress, 32.2 MPa von Mises stress, and 42% effective strain) [22].

## 4. Discussion

In the present study, we examined the sensitivity of polyethylene stresses in the LTJR to coronal and axial plane misalignment at L5-S1. We studied the polyethylene stresses as a proxy for surface damage and wear in this design, having established the elevated stresses that were associated with benchtop wear and impingement studies [22]. Although we found that the polyethylene stresses were generally higher at L5-S1 than our previous findings at L4-L5 [23], the L5-S1 values remained well below the levels of impingement studies [21,22], indicating that they were reasonably low from a biomedical engineering perspective. We likewise found that contact pressure and von Mises stress were relatively insensitive (varying < 20% from baseline) to coronal and axial plane misalignment, similar to our previous findings at L4-L5 [23]. Effective strain was more sensitive to coronal misalignment, increasing from 4.8% at baseline to a maximum of 9.9%, although it remained well below the level associated with Mode IV impingement (42%).

Biomechanically, the L5-S1 segment poses distinct challenges compared to other lumbar levels. The sacral slope increases the anterior shear forces generated by body weight, while its inferior location creates the longest possible moment arm during forward bending, producing the highest compressive loads in the lumbar spine. As a result, L5-S1 experiences the greatest overall biomechanical loading, particularly in flexion. Notably, despite the elevated loading conditions applied in the present study, the resulting contact pressures and internal stresses and strains remained within acceptable limits when compared to other joint replacement technologies, with strain values more similar to knee devices than hip devices [39,40,41,42]. However, finite element analyses of knee and hip polyethylene components have shown comparatively large increases in stress and strain due to implant positioning [41,42,43].

From a design verification standpoint, assessing the LTJR device implanted at the most biomechanically demanding lumbar level, L5-S1, represents a conservative approach for evaluating polyethylene stress sensitivity to misalignment under the forward bending condition studied. Thus, the primary contribution of this work is the robustness of its design evaluation. The finding that polyethylene stresses remain well below impingement thresholds at L5-S1, even during flexion and under misalignment conditions, provides evidence that the LTJR device’s conforming dual ball-and-socket bearing design can effectively tolerate the positional variability expected during implantation. Alongside our previous analyses at the wear simulator level [22], at L4-L5 [23], and under aggressive loading at L5-S1 [32], the present study adds to a progressive credibility evaluation consistent with the ASME V&V 40 [30], which may support future regulatory submissions incorporating in silico evidence as a complement to benchtop and clinical data.

In traditional anterior disc replacement technologies, the facet joints are generally left intact. At first glance, this may appear biomechanically advantageous, as these structures can continue to help resist anterior shear forces. However, prior studies [44,45] have shown that the altered intervertebral biomechanics introduced by a disc replacement’s bearing surfaces can lead to non-conformance (i.e., lift-off) of the contacting polyethylene surfaces. This loss of congruency increases localized contact stresses, which in turn accelerates polyethylene wear and elevates the risk of device failure. The LTJR device is specifically designed to be used in the absence of the facet joints. Consequently, the device is responsible for resisting anterior shear and axial rotation.

As demonstrated by the data, even during forward bending conditions, the contact pattern remains congruous and demonstrates full conformance of the bearing surfaces. As a result, the contact pressure and internal stresses and strains remain reasonably low. Therefore, the data from the current study demonstrates that a LTJR device, without any existing facet joints, with multiple conforming bearing surfaces, sufficiently resists the biomechanical challenges of forward bending at L5-S1. Further, this behavior is maintained across a range of positional variability defined as clinically reasonable in the surgical technique guide. Among the three parameters studied, coronal tilt produced the largest changes in polyethylene stress and shifts in contact pattern away from the dome center, suggesting that achieving accurate coronal alignment may deserve the greatest intraoperative attention. This effect was also directionally asymmetric, with positive tilt shifting the contact and reducing its area more than negative tilt, thereby raising peak stress and strain. Because this asymmetry was less pronounced in our earlier L3-L5 and spine wear simulator models, it likely depends on the specific anatomy rather than on the bearing design alone. Increased unevenness and concavity of the vertebral endplates relative to those in the previous L3-L5 model [23,29] is one plausible contributor; however, a multi-anatomy study would be needed to confirm whether the effect is governed more so by the specific model anatomy or the lumbar level more broadly. Regardless, the small magnitude of these differences, along with the insensitivity of polyethylene stresses to convergence angle and AP offset, demonstrates the LTJR’s robustness against inherent intraoperative placement variability, even at the most biomechanically demanding lumbar level.

Surgery utilizing the MOTUS^®^ procedure and device at L5-S1 is unique from other lumbar levels and requires special considerations. First, the exposure, whether midline or through a paramedian approach, should not include exposure of the transverse process of L5. The iliolumbar ligament from the pelvis to the transverse process of L5 is a primary restraint against anterior shear translation of L5. Dissecting and exposing the transverse process risks inadvertent release of this important ligament. For this reason, it is recommended to use a midline approach at this level rather than paramedian. The paramedian approach does have some advantages due to the need to place the device parallel with and following the converging pedicular angles. Secondly, there should be a careful review of the distance from the inferior pedicle of L5 to the L5 vertebral body inferior endplate. The L5 nerve root is just medial and inferior to the pedicle as it is exiting the foramen. Particularly in cases of L5 lytic spondylolisthesis, there is often significant wedging of the L5 vertebral body height less posteriorly than anteriorly. The smaller posterior height of the L5 vertebral body can narrow the space available for the exiting nerve root. The average diameter for the L5 exiting nerve root is 4 mm [46]; thus, it is recommended to have approximately 6 mm of space between the inferior border of the L5 pedicle and the top of the L5 inferior endplate. This distance can be best estimated pre-operatively on a CT scan. If there is less than the desired space, the surgeon needs to use a high-speed burr to carry out a 10–15% infero-medial pedicloplasty. The pedicloplasty will create an additional 2–3 mm of space to allow a superior positioning of the exiting nerve root. This same technique can be used at other levels to create additional space for the exiting nerve, although it is rarely used cranial to L5.

Our study had some limitations. First, the L4-S1 model was based on a single patient anatomy and does not capture variability in characteristics like vertebral geometry, sacral slope, or degenerative changes. The influence of these parameters on segmental biomechanics could affect the sensitivity of polyethylene stresses to misalignment, and future work should examine a range of patient anatomies to assess the generalizability of our findings. It is expected that sacral slope and segmental lordosis would influence the orientation of the compressive and shear load vectors at L5-S1 [47,48], while vertebral geometry and degenerative changes, which are known to influence facet joint forces and intervertebral disc pressure [49,50], would alter local load transfer. While the model incorporates CT-based anatomy from a representative IDE study patient, an important advancement over our previous FEM approach [23], it was developed to support a parametric analysis of implant placement rather than patient-specific simulations, which would require additional validation activities. Nevertheless, our approach may be extended to examine additional patients in a future in silico study. Second, our research question was limited to the parametric analysis of reasonable misalignment and not gross malalignment, subsidence, or migration. Third, we did not simulate the combined, multivariate effects of misalignment in the axial and coronal planes as part of this analysis; however, our univariate approach allows us to understand the relative sensitivity of the device to specific misalignment boundary conditions. Fourth, the boundary conditions explored for this analysis were limited to forward bending at the L5-S1 level. Although this is among the most common and mechanically challenging postures for the lumbar spine, and the use of this singular loading scenario helps isolate the effect of each misalignment parameter under a common, well-defined loading condition, it excludes axial rotation, lateral bending, extension, and combined motions. Aggressive loading scenarios such as these were investigated in a separate study [32]. Fifth, the bone–implant interface was modeled to reflect ideal fixation and does not account for potential micromotion, subsidence, or loosening that may occur in vivo. In practice, imperfect fixation or endplate settling could alter load transfer and the biomechanical effects of misalignment. Other modeling simplifications, while standard in computational design verification, may further contribute to differences between the predicted stress and strain values and those encountered in vivo. Despite these limitations, we judged the model to be credible for addressing our research question based on the guidance provided by ASME V&V 40 [30].

## 5. Conclusions

This study concludes our research into the sensitivity of LTJR to coronal and axial plane misalignment in the lumbosacral spine. Across all eight misalignment scenarios, we found that contact pressure and von Mises stress varied by <20% from baseline values at L5-S1 and were comparable to our findings at L4-L5, whereas effective strain was more sensitive to coronal tilt, increasing from 4.8% to 9.9% at +20 degrees. Among the parameters evaluated, coronal tilt produced the largest stress variations, while convergence angle and AP offset had minimal effects. Furthermore, peak contact pressures, stresses, and strains remained below the levels associated with impingement-mode wear during previous bench testing. Additionally, the results demonstrate that the conforming dual ball-and-socket bearing design of the LTJR maintains surface congruency under a range of positions at L5-S1, the most biomechanically demanding segment in the lumbar spine. Within the misalignment magnitudes and loading conditions modeled here, this tolerance suggests an implant design that can accommodate the inherent variability of intraoperative placement without substantial alteration of the bearing’s mechanical environment. Together with our prior analyses (i.e., wear simulator and implantation at L4-L5), these findings extend the credibility of our computational modeling framework across the two most commonly treated levels in the IDE study. This progressive body of evidence provides a foundation for future patient-specific finite element models that can, with further validation, be sufficiently credible for in silico clinical studies.

## Figures and Tables

**Table 1:** Peak contact pressure values (MPa) for the current FEM, the L3-L5 FEM, and the wear simulator FEM.

Case	Current Study (% Change from Baseline)	L3-L5 FEM [23]	Simulator (Mode I) FEM [22]
Baseline	35	29.9	32.5
Case 1: 30 Degrees Convergence	34.0 (−2.9)	30.7	33.7
Case 2: 20 degrees Convergence	32.5 (−7.1)	30.3	38.2
Case 3: −2 mm A-P Offset	35.9 (2.6)	30.7	36.4
Case 4: 4 mm A-P Offset	36.6 (4.6)	33.0	37.0
Case 5: −20 Degrees Coronal Tilt	28.2 (−19.4)	32.2	40.5
Case 6: −10 Degrees Coronal Tilt	28.8 (−17.7)	30.0	33.3
Case 7: 10 Degrees Coronal Tilt	36.5 (4.3)	31.1	35.7
Case 8: 20 Degrees Coronal Tilt	36.4 (4.0)	36.7	41.6

## Data Availability

The original contributions presented in this study are included in the article/Supplementary Material. Further inquiries can be directed to the corresponding author.

## Supplementary Materials

The following supporting information can be downloaded at: https://www.mdpi.com/article/10.3390/bioengineering13090987/s1, Table S1: Peak von Mises stress values (MPa) for the current FEM, the L3-L5 FEM, and the wear simulator FEM; Table S2: Peak effective strain values for the current FEM, the L3-L5 FEM, and the wear simulator FEM; Figure S1: Von Mises stress plots at maximum loading during bending over for the baseline, 30 degrees, and 20 degrees convergence angle simulations for the L4-S1 FEM; Figure S2: Effective strain plots at maximum loading during bending over for the baseline, 30 degrees, and 20 degrees convergence angle simulations for the L4-S1 FEM; Figure S3: Von Mises stress plots at maximum loading during bending over for the baseline, 2 mm, and 4 mm AP offset simulations for the L4-S1 FEM; Figure S4: Effective strain plots at maximum loading during bending over for the baseline, 2 mm, and 4 mm AP offset simulations for the L4-S1 FEM; Figure S5: Von Mises Stress plots at maximum loading during bending over for the baseline, −20 degree, −10 degree, 10 degree, and 20 degree coronal tilt simulations for the L4-S1 FEM; Figure S6: Effective strain plots at maximum loading during bending over for the baseline, −20 degree, −10 degree, 10 degree, and 20 degree coronal tilt simulations for the L4-S1 FEM. References [22,23] are cited in the Supplementary Materials.

## References

[1] Hashimoto K., Aizawa T., Kanno H., Itoi E. (2019). Adjacent segment degeneration after fusion spinal surgery—A systematic review. Int. Orthop..

[2] Pellisé F., Hernández A., Vidal X., Minguell J., Martínez C., Villanueva C. (2007). Radiologic assessment of all unfused lumbar segments 7.5 years after instrumented posterior spinal fusion. Spine.

[3] Schulte T.L., Leistra F., Bullmann V., Lerner T., Liljenqvist U., Hackenberg L. (2007). Disc Height Reduction in Adjacent Segments and Clinical Outcome 10 years after Lumbar 360° Fusion. Eur. Spine J..

[4] Cunningham B.W., Gordon J.D., Dmitriev A.E., Hu N., McAfee P.C. (2003). Biomechanical evaluation of total disc replacement arthroplasty: An in vitro human cadaveric model. Spine.

[5] Auerbach J.D., Wills B.P., McIntosh T.C., Balderston R.A. (2007). Evaluation of spinal kinematics following lumbar total disc replacement and circumferential fusion using in vivo fluoroscopy. Spine.

[6] Salzmann S.N., Plais N., Shue J., Girardi F.P. (2017). Lumbar disc replacement surgery—Successes and obstacles to widespread adoption. Curr. Rev. Musculoskelet. Med..

[7] Panjabi M., Henderson G., Abjornson C., Yue J. (2007). Multidirectional testing of one-and two-level ProDisc-L versus simulated fusions. Spine.

[8] Kurtz S.M., Peloza J., Siskey R., Villarraga M.L. (2005). Analysis of a retrieved polyethylene total disc replacement component. Spine J..

[9] Choma T.J., Miranda J., Siskey R., Baxter R.M., Steinbeck M.J., Kurtz S.M. (2008). Retrieval analysis of a ProDisc-L total disc replacement. Clin. Spine Surg..

[10] Kafer W., Clessienne C.B., Daxle M., Kocak T., Reichel H., Cakir B. (2008). Posterior component impingement after lumbar total disc replacement: A radiographic analysis of 66 ProDisc-L prostheses in 56 patients. Spine.

[11] Veruva S.Y., Lanman T.H., Isaza J.E., Freeman T.A., Kurtz S.M., Steinbeck M.J. (2017). Periprosthetic UHMWPE Wear Debris Induces Inflammation, Vascularization, and Innervation After Total Disc Replacement in the Lumbar Spine. Clin. Orthop. Relat. Res..

[12] Punt I.M., Visser V.M., van Rhijn L.W., Kurtz S.M., Antonis J., Schurink G.W., van Ooij A. (2008). Complications and reoperations of the SB Charite lumbar disc prosthesis: Experience in 75 patients. Eur. Spine J..

[13] van Ooij A., Kurtz S.M., Stessels F., Noten H., van Rhijn L. (2007). Polyethylene wear debris and long-term clinical failure of the Charite disc prosthesis: A study of 4 patients. Spine.

[14] van Ooij A., Oner F.C., Verbout A.J. (2003). Complications of artificial disc replacement: A report of 27 patients with the SB Charite disc. Spine.

[15] Baxter R.M., Macdonald D.W., Kurtz S.M., Steinbeck M.J. (2013). Severe impingement of lumbar disc replacements increases the functional biological activity of polyethylene wear debris. J. Bone Jt. Surg..

[16] Siepe C.J., Wiechert K., Khattab M.F., Korge A., Mayer H.M. (2007). Total lumbar disc replacement in athletes: Clinical results, return to sport and athletic performance. Eur. Spine J..

[17] National Library of Medicine (U.S.) (2025). MOTUS Total Joint Replacement Investigational Device Exemption Study. ClinicalTrials.gov. https://clinicaltrials.gov/study/NCT05438719.

[18] Goldstein J.A., Nunley P.D., Sivaganesan A., Sielatycki J.A., Jorgensen A.Y., Khachatryan A., Humphreys S.C., Block J.E., Hodges S.D., Nel L.J. (2025). Total Joint Replacement of the Lumbar Spine: The Future of Motion Preservation. Int. J. Spine Surg..

[19] Humphreys S.C., Block J.E., Sivaganesan A., Nel L.J., Peterman M., Hodges S.D. (2025). Optimizing the clinical adoption of total joint replacement of the lumbar spine through imaging, robotics and artificial intelligence. Expert Rev. Med. Devices.

[20] Sielatycki A.J., Devin C.J., Pennings J., Koscielski M., Metcalf T., Archer K.R., Dunn R., Craig Humphreys S., Hodges S. (2021). A novel lumbar total joint replacement may be an improvement over fusion for degenerative lumbar conditions: A comparative analysis of patient-reported outcomes at one year. Spine J..

[21] Siskey R.L., Yarbrough R.V., Spece H., Hodges S.D., Humphreys S.C., Kurtz S.M. (2023). In Vitro Wear of a Novel Vitamin E Crosslinked Polyethylene Lumbar Total Joint Replacement. Bioengineering.

[22] Kurtz S.M., Rundell S.A., Spece H., Yarbrough R. (2025). Sensitivity of Lumbar Total Joint Replacement Contact Stresses Under Misalignment Conditions—Finite Element Analysis of a Spine Wear Simulator. Bioengineering.

[23] Rundell S., Kurtz S., Spece H., Goldstein J., Hodges S., Yarbrough R. (2025). Sensitivity of Lumbar Total Joint Replacement to Axial and Coronal Plane Misalignment Using Computational Modeling. Int. J. Spine Surg..

[24] Sabnis A.B., Chamoli U., Diwan A.D. (2018). Is L5–S1 motion segment different from the rest? A radiographic kinematic assessment of 72 patients with chronic low back pain. Eur. Spine J..

[25] Wang K., Deng Z., Chen X., Shao J., Qiu L., Jiang C., Niu W. (2023). The Role of Multifidus in the Biomechanics of Lumbar Spine: A Musculoskeletal Modeling Study. Bioengineering.

[26] Ramakrishna V.A., Chamoli U., Viglione L.L., Tsafnat N., Diwan A.D. (2018). The role of sacral slope in the progression of a bilateral spondylolytic defect at L5 to spondylolisthesis: A biomechanical investigation using finite element analysis. Glob. Spine J..

[27] Oxland T.R. (2016). Fundamental biomechanics of the spine—What we have learned in the past 25 years and future directions. J. Biomech..

[28] Kurtz S.M., Rundell S.A., Spece H., Siskey R.L., Yarbrough R.V. (2026). High demand loading conditions and their effect on polyethylene stresses in lumbar total joint replacement: Implications for spine wear protocols. J. Orthop. Res.^®^.

[29] Rundell S.A., Spece H., Yarbrough R.V., Kurtz S.M. (2026). Polyethylene stresses in lumbar total joint replacement under elevated loading: Insights from an anatomic finite element model. Bioengineering.

[30] (2018). Assessing Credibility of Computational Modeling Through Verification and Validation: Application to Medical Devices.

[31] United States Food and Drug Administration (2023). Credibility of Computational Modeling and Simulation in Medical Device Submissions: Guidance for Industry and Food and Drug Administration Staff.

[32] Rundell S., Spece H., Yarbrough R., Kurtz S. (2026). The Effect of High Demand Loading Conditions on Polyethylene Stresses in Total Joint Replacement of the Lumbar Spine at L5-S1. Ann. Biomed. Eng..

[33] Pieper S., Haouchine N., Hackney D.B., Wells W.M., Sanhinova M., Balboni T., Spektor A., Huynh M., Tanguturi S., Kim E. (2024). Spine Metastatic Bone Cancer: Pre and Post Radiotherapy CT (Spine-Mets-CT-SEG) Dataset Version 1.

[34] Le Huec J.-C., Thompson W., Mohsinaly Y., Barrey C., Faundez A. (2019). Sagittal balance of the spine. Eur. Spine J..

[35] Chaffin D.B. (1998). Prediction of Population Strengths.

[36] Chaffin D.B., Erig M. (1991). Three-dimensional biomechanical static strength prediction model sensitivity to postural and anthropometric inaccuracies. IIE Trans..

[37] Rossman S., Meyer E., Rundell S. (2022). Development of a finite element lumbar spine model to predict intervertebral disc herniation risk factors. Comput. Methods Biomech. Biomed. Eng..

[38] White A. (1990). Clinical Biomechanics of the Spine.

[39] Giddings V.L., Kurtz S.M., Edidin A.A. (2001). Total knee replacement polyethylene stresses during loading in a knee simulator. J. Tribol..

[40] Bartel D.L., Rawlinson J., Burstein A., Ranawat C., Flynn W. (1995). Stresses in polyethylene components of contemporary total knee replacements. Clin. Orthop. Relat. Res..

[41] Huang C.-H., Lu Y.-C., Hsu L.-I., Liau J.-J., Chang T.-K., Huang C.-H. (2020). Effect of material selection on tibial post stresses in posterior-stabilized knee prosthesis: A comparison among conventional, cross-linked, and vitamin E-stabilized polyethylene. Bone Jt. J..

[42] Hua X., Li J., Wang L., Jin Z., Wilcox R., Fisher J. (2014). Contact mechanics of modular metal-on-polyethylene total hip replacement under adverse edge loading conditions. J. Biomech..

[43] Liau J.-J., Cheng C.-K., Huang C.-H., Lo W.-H. (2002). The effect of malalignment on stresses in polyethylene component of total knee prostheses–a finite element analysis. Clin. Biomech..

[44] Rundell S., Day J., Siskey R., Kurtz S., MacDonald D., Isaza J. (2011). Derivation of clinically relevant boundary conditions suitable for evaluation of chronic impingement of lumbar total disk replacement: Application to standard development. J. ASTM Int..

[45] Rundell S., Day J., Isaza J., Guillory S., Kurtz S. (2012). Lumbar total disc replacement impingement sensitivity to disc height distraction, spinal sagittal orientation, implant position, and implant lordosis. Spine.

[46] Chaves H., Bendersky M., Goñi R., Gómez C., Carnevale M., Cejas C. (2018). Lumbosacral plexus root thickening: Establishing normal root dimensions using magnetic resonance neurography. Clin. Anat..

[47] Bassani T., Casaroli G., Galbusera F. (2019). Dependence of lumbar loads on spinopelvic sagittal alignment: An evaluation based on musculoskeletal modeling. PLoS ONE.

[48] Chen J., Hume P.A., Wyatt H., Yeung T., Choisne J. (2025). Understanding the effect of lumbar lordosis angle on vertebral load distribution during walking. Comput. Methods Biomech. Biomed. Eng..

[49] Putzer M., Ehrlich I., Rasmussen J., Gebbeken N., Dendorfer S. (2016). Sensitivity of lumbar spine loading to anatomical parameters. J. Biomech..

[50] Knapik G.G., Mendel E., Bourekas E., Marras W.S. (2026). Impact of vertebrae shape variation on lumbar spine loading: An image-based computational modeling study. Eur. Spine J..

