# Supplementary material for: Effect of Axial and Coronal Malalignment on Polyethylene Stresses in Lumbosacral Total Joint Replacement: A Finite Element Study
Source: Bioengineering (Basel). 2026 Aug 27;13(9):987. doi: 10.3390/bioengineering13090987 (PMC13603936; doi:10.3390/bioengineering13090987)
Supplement: Supplementary file 1 [file bioengineering-13-00987-s001.zip › bioengineering-4485284-supplementary.pdf]

Supplementary Materials

Table S1. Peak von Mises stress values (MPa) for the current FEM, the L3-L5 FEM, and the wear simulator FEM.

Case	Current Study (% change from baseline)	L3-L5 FEM(Rundell et al., 2025)	Simulator (Mode I) FEM(Kurtz et al., 2025)
Baseline	19.9	21.9	21.0
Case 1: 30 Degrees Convergence	19.7 (−1.0)	21.9	21.1
Case 2: 20 degrees Convergence	19.7 (−1.0)	21.7	21.3
Case 3: 2 mm A-P Offset	20.1 (1.0)	22.1	21.6
Case 4: 4 mm A-P Offset	20.9 (5.0)	23.2	22.5
Case 5: −20 Degrees Coronal Tilt	19.3 (−3.0)	18.7	23.9
Case 6: −10 Degrees Coronal Tilt	19.4 (−2.5)	20.6	23.1
Case 7: 10 Degrees Coronal Tilt	20.9 (5.0)	22.4	22.6
Case 8: 20 Degrees Coronal Tilt	22.4 (12.6)	25.1	23.8

Table S2. Peak effective strain values for the current FEM, the L3-L5 FEM, and the wear simulator FEM.

Case	Current Study (% change from baseline)	L3-L5 FEM(Rundell et al., 2025)	Simulator (Mode I) FEM
Baseline	4.8	8.4	7.1
Case 1: 30 Degrees Convergence	4.6 (−4.2)	9.5	7.4
Case 2: 20 degrees Convergence	4.4 (−8.3)	8.9	7.6
Case 3: 2 mm A-P Offset	5.6 (16.7)	11.3	10.5
Case 4: 4 mm A-P Offset	6.6 (37.5)	16.7	11.6
Case 5: −20 Degrees Coronal Tilt	4.1 (−14.6)	4.4	18.3
Case 6: −10 Degrees Coronal Tilt	4.2 (−12.5)	6.0	13.5
Case 7: 10 Degrees Coronal Tilt	6.8 (41.7)	9.7	11.4
Case 8: 20 Degrees Coronal Tilt	9.9 (106.3)	16.9	17.1

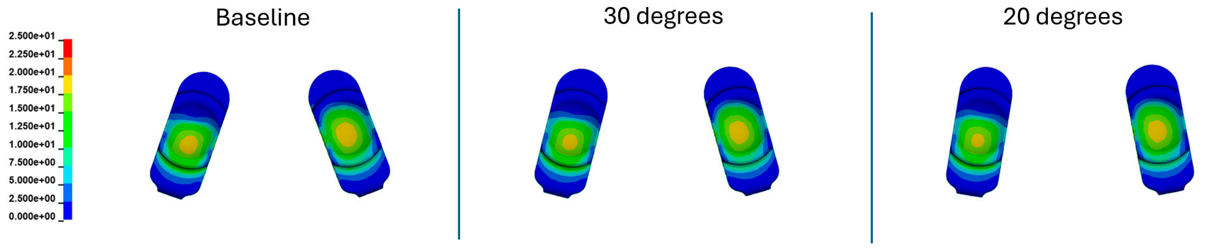

Figure S1. Von Mises stress plots at maximum loading during bending over for the baseline, 30 degrees, and 20 degrees convergence angle simulations for the L4-S1 FEM.

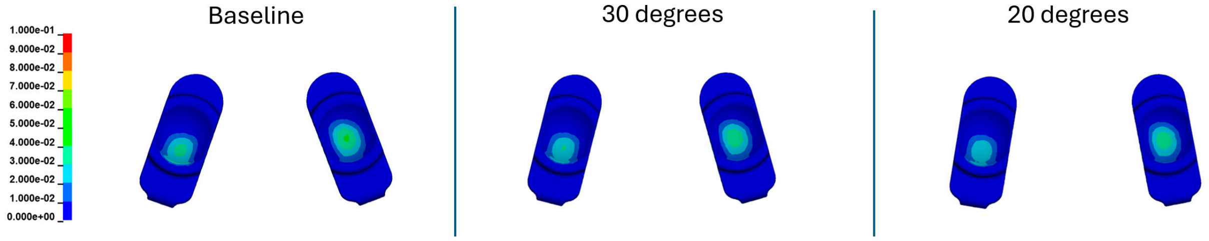

Figure S2. Effective strain plots at maximum loading during bending over for the baseline, 30 degrees, and 20 degrees convergence angle simulations for the L4-S1 FEM.

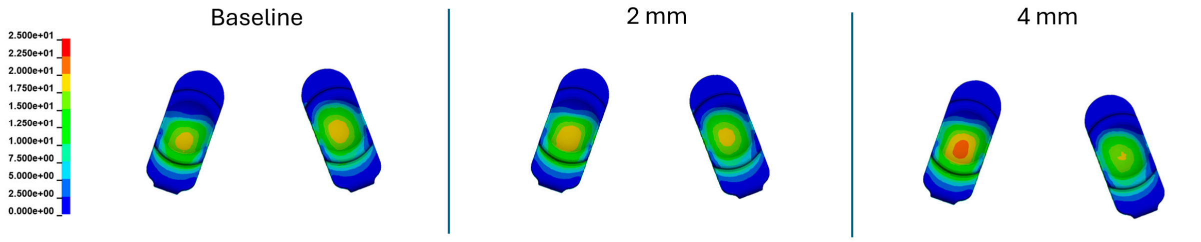

Figure S3. Von Mises stress plots at maximum loading during bending over for the baseline, 2 mm, and 4 mm AP offset simulations for the L4-S1 FEM.

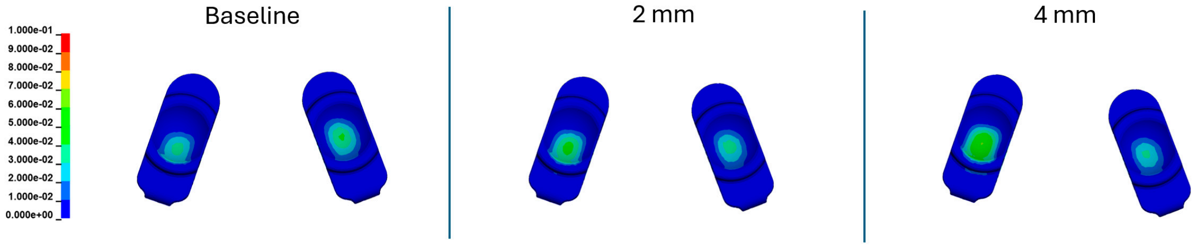

Figure S4. Effective strain plots at maximum loading during bending over for the baseline, 2 mm, and 4 mm AP offset simulations for the L4-S1 FEM.

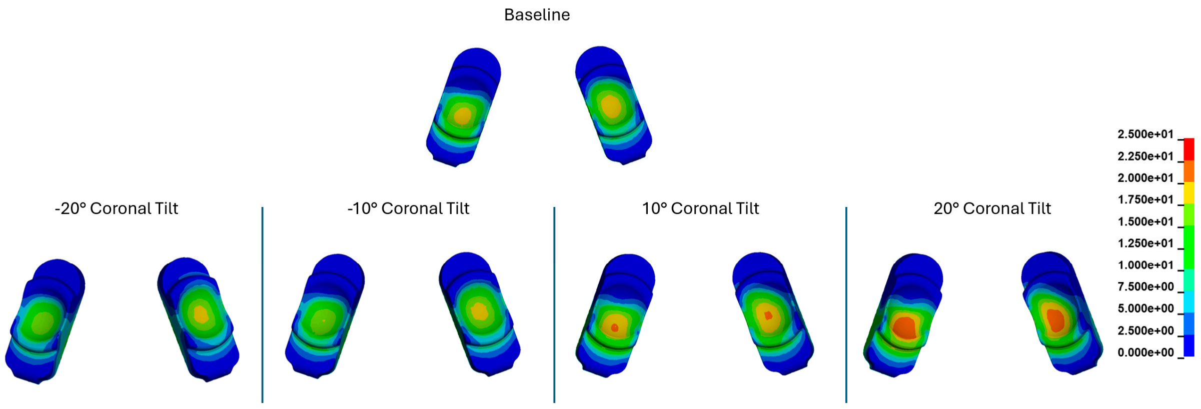

Figure S5. Von Mises Stress plots at maximum loading during bending over for the baseline, −20 degree, −10 degree, 10 degree, and 20 degree coronal tilt simulations for the L4-S1 FEM.

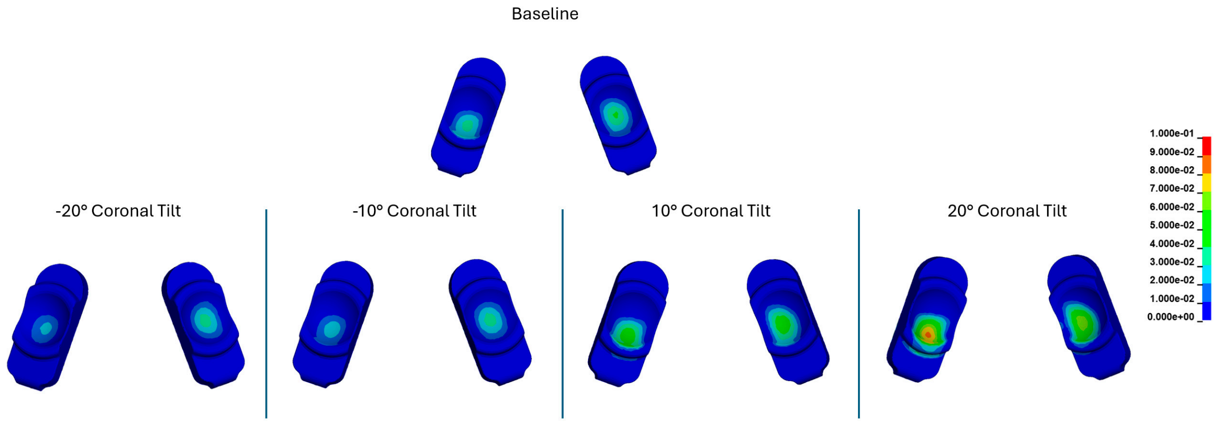

Figure S6. Effective strain plots at maximum loading during bending over for the baseline, −20 degree, −10 degree, 10 degree, and 20 degree coronal tilt simulations for the L4-S1 FEM.

References

22. Kurtz, S.M.; Rundell, S.A.; Spece, H.; Yarbrough, R. Sensitivity of Lumbar Total Joint Replacement Contact Stresses Under Misalignment Conditions—Finite Element Analysis of a Spine Wear Simulator. Bioengineering 2025, 12, 229.

23. Rundell, S.; Kurtz, S.; Spece, H.; Goldstein, J.; Hodges, S.; Yarbrough, R. Sensitivity of Lumbar Total Joint Replacement to Axial and Coronal Plane Misalignment Using Computational Modeling. Int. J. Spine Surg. 2025, 19, 635.
